# Training of Community Health Workers in Diabetes Lead to Improved Outcomes for Diabetes Screening and Management in Low- and Middle-Income Countries: Protocol for a Systematic Review

**DOI:** 10.2196/57313

**Published:** 2024-08-21

**Authors:** Anirudh Gaurang Gudlavalleti, Giridhara R Babu, Sureshkumar Kamalakannan, G V S Murthy, Nicolaas C Schaper, Onno C P van Schayck

**Affiliations:** 1 Pragyaan Sustainable Health Outcomes Foundation Hyderabad India; 2 Department of Family Medicine Care and Public Health Research Institute Maastricht University Maastricht Netherlands; 3 Qatar University Doha Qatar; 4 Department of social work, education and community wellbeing Northumbria University Newcastle Upon Tyne United Kingdom

**Keywords:** community health workers, diabetes training, community health worker training, systematic review, diabetes management, community training, low-and-middle-income countries, diabetes, type 2 diabetes mellitus, prevalence, rural, health care professionals, barriers

## Abstract

**Background:**

Diabetes is a growing concern worldwide, particularly in low- and middle-income countries (LMICs). Type 2 diabetes mellitus constitutes a significant proportion of cases and is associated with debilitating microvascular complications. Type 2 diabetes mellitus is steadily increasing among the LMICs where many barriers to health care exist. Thus, task shifting to community health workers (CHWs) has been proposed as a solution to improve diabetes management in these settings. However, CHWs often lack the necessary training to manage diabetes effectively. Thus, a systematic review is required to present evidence of the highest degree for this intervention.

**Objective:**

This study aims to establish the protocols for a systemic review.

**Methods:**

Using the Participants Intervention Comparator Outcome Time Study Design (PICOTS) framework, this study outlines a systematic review aiming to evaluate the impact of training programs for CHWs in diabetes management in LMICs. Quantitative studies focusing on CHWs, diabetes training, focusing on diabetes management outcomes like hemoglobin A_1c_ levels and fasting blood glucose levels, between January 2000 and December 2023 and found on databases such as PubMed, Ovid MEDLINE, Evidence Based Medicine Reviews, BASE, Google Scholar, and Web of Science will be included. We will include randomized controlled trials but will also include observational studies if we find less than 5 randomized controlled trials. An author committee consisting of 3 reviewers will be formed, where 2 reviewers will conduct the review independently while the third will resolve all disputes. The Cochrane Methods Risk of Bias Tool 2 will be used for assessing the risk of bias and the Grading of Recommendations, Assessment, Development and Evaluation approach for the meta-analysis and narrative synthesis analysis will be used. The results will be presented in a PRISMA (Preferred Reporting Items for Systematic Reviews and Meta-Analyses) diagram.

**Results:**

The review will begin in May 2024 and conclude in 3 months.

**Conclusions:**

The review will synthesize existing evidence and provide insights into the effectiveness of such programs, informing future research and practice in diabetes care in LMICs.

**Trial Registration:**

PROSPERO CRD42022341717; https://tinyurl.com/jva2hpdr

**International Registered Report Identifier (IRRID):**

PRR1-10.2196/57313

## Introduction

Diabetes is a global epidemic that is threatening to grow exponentially and impact the lives of those affected. The International Diabetes Federation estimates that 463 million people will have diabetes globally and projects this number increasing to 700 million by 2045 [1]. Almost 80% of the world’s patients with diabetes reside in low- and middle-income countries (LMICs) [2]. Type 2 diabetes mellitus (T2DM) constitutes a majority of patients with diabetes (almost 90%) and it severely affects them by causing microvascular complications that are often debilitating and morbid [3]. The condition itself is a risk factor for many other conditions such as stroke, cancer, and tuberculosis and causes damage to critical organs such as the heart, kidneys, eyes, and the limbs [4,5]. The immunocompromised nature of the patients makes them susceptible to infections and overall contributes to poorer health outcomes.

The LMICs are also gradually witnessing a rise in the prevalence of the disease in rural areas [6]. The LMICs are also plagued with barriers like availability of trained health care professionals who are required to cater to this growing number of patients with diabetes, long distances to the health centers, and lack of transportation facilities which often cause poorer uptake of health services [7-9].

The T2DM disease burden has increased exponentially in LMICs due to continuous upward growth in the trajectory of common risk factors responsible for T2DM. These have caused an increase in the comorbidities associated with the T2DM condition across the LMICs [10]. Similarly, the age-standardized death rates over the past decade have seen a 2- to 3-fold increase in the LMICs, when compared to the upper- or middle-income countries [11]. Recent studies have shown an increase in the prevalence of T2DM in lower socioeconomic areas of LMICs [12,13]. They also show that the increase in the prevalence of prediabetes is greater in these areas than the rate of increase in the urban areas [6,14]. Hence, the burden is set to increase among populations that have remote access to health care services or cannot afford the life-long management costs associated with T2DM [8,12,13,15,16].

Thus, a potential method of overcoming these barriers has been the use of community health workers (CHWs) for task shifting in LMICs for both infectious diseases and noncommunicable diseases [17-24]. All such task shifting initiatives need to ensure that the participating CHWs are provided with requisite training. Most CHW studies have found them to be deficient in knowledge and associated skills, and hence training them for any task-shifting initiative is critical for its success [25-28].

Where training has been provided, the results have generally been encouraging. However, such training programs have been few, and their evaluations have been rare. Evidence is found wanting for the efficiency of trained CHWs in LMICs in screening and managing patients with diabetes. Hence, a systematic review is required to present evidence of the highest degree for this intervention, and we propose to conduct the systematic review to understand if training of CHWs in diabetes would lead to improved outcomes for diabetes screening and management in LMICs.

Thus, the objective of this paper is to establish the protocols for the systematic review.

The systematic review has been registered with PROSPERO at PROSPERO 2022 (CRD42022341717).

## Methods

### Study Design

The criteria for considering studies for the systematic review would be done according to Participants Intervention Comparator Outcome Time Study Design (PICOTS) framework. Therefore, we would be considering the studies under the following conditions, the participants, the intervention, the comparators, the outcomes, the types of studies, and the timing of the study. Along with these, the databases to be used for the search will also be considered.

### Description of Condition

T2DM will be the condition of interest. It accounts for almost 90% of diabetes globally and thereby affects a larger proportion of the population suffering from it. We will consider the World Health Organization (WHO) cutoff value of 126 mg/dl for fasting plasma glucose or 200 mg/dl for 2-hour post food plasma glucose to classify a person with diabetes [29].

### Description of Participants

CHWs of LMICs will be considered participants for the studies or trials to be included in this review. The WHO defines CHWs to be “members of the communities where they work, should be selected by the communities, should be answerable to the communities for their activities, should be supported by the health system but not necessarily be a part of its organization, and have shorter training than professional workers” [30]. Hence, we will include all studies with CHWs in LMICs. The LMICs will be defined according to the World Bank definition of economies as those with a gross national income per capita between US $1136 and US $4465, calculated using the World Bank Atlas method [31]. A detailed list of the countries is given in Multimedia Appendix 1.

### Description of the Intervention

The CHWs employed for task shifting have been found to have deficient knowledge. This can be corrected by providing them with the requisite training as that has been evidenced as an effective instrument for improving their knowledge, skills, and practices in LMICs [32,33]. Training in T2DM management includes the systematic application of formal processes to impart knowledge and help people acquire the skills necessary for them to perform their jobs satisfactorily. Hence, studies reporting any training related to the knowledge, skills and performance of the CHWs will be included. Some of the common aspects of training might include the screening of glycemia, lifestyle advice and education for patients, measurement of glycemia, and referral indications related to the aforementioned items.

### Description of the Comparator

Standard training given to CHWs will be considered as the comparator. Therefore, any such training (whether focused on diabetes or even no training) provided to the CHWs would be included as the comparator.

### Description of Studies

For the review, we will include randomized controlled trials (RCTs) as the first choice of studies, conducted between January 2000 to December 2023, in LMICs and published in English. This is due to the fact they present the highest degree of scientific evidence, and WHO and the World Medical Association published their guidelines and resolutions on task shifting respectively [34,35] around this time period. If there are less than 5 such studies, we will then include observational studies as well. For high scientific evidence, we will exclude qualitative studies. The included studies will focus on the training of CHWs in diabetes and look at diabetes management as their outcome. The details about the outcomes are detailed in the Description of Outcomes section.

### Description of Outcomes

As we wish to study the effect of the training on diabetes management, we will include studies reporting any of the following outcomes with the respective measures of effect: (1) referral for diabetes (change in the number of referrals), (2) screening for diabetes (change in the number of screenings), (3) hemoglobin A_1c_ (HbA_1c_) values (change in the mean HbA_1c_ values), and (4) fasting blood glucose values (change in the mean fasting blood glucose values).

### Databases and Other Sources

For our review, we would search for appropriate studies on the following databases: Ovid MEDLINE, PubMed, Scopus, Cochrane Central, and Evidence Based Medicine Reviews. Additionally, we will also search the bibliographies of all the trials included based on our search strategies to identify further relevant studies. We will also be searching for all pertinent grey literature as this will help us identify the case studies and reports in greater detail. This will include a search of the databases about gray literature such as BASE, Google Scholar, WHO International Clinical Trials Registry Platform, and WorldCat. These will help us identify reports of studies conducted by private and civil society organizations or by other PhD students. The provisional search strategy for PubMed has been described in Multimedia Appendix 2.

### Study Selection for the Review

Study titles and abstracts, which will be obtained through electronic source search, will be independently read by the reviewers (AGG and RD). The papers would then be selected for eligibility, based on the criteria. Papers that the authors find to be eligible will be retrieved in the full-text form. In case any of these papers are later found to be unsuitable for inclusion in our review, they would be mentioned separately in detail in a tabular manner, titled table of excluded studies. The PRISMA-P (Preferred Reporting Items for Systematic Reviews and Meta-Analyses–Protocols) [36] guidelines have been adhered to for this protocol and PRISMA (Preferred Reporting Items for Systematic Reviews and Meta-Analyses) guidelines will be adhered to for the conduct of the review (Multimedia Appendix 3). The full-text review will be carried out independently by 2 reviewers (AGG and RD). The third reviewer (GRB) will resolve any disagreements, which might arise.

### Data Extraction and Synthesis

The data extraction will be done by 2 reviewers independently. They will first develop a custom-made data extraction form using the Cochrane collaboration template for RCTs and non-RCTs. This form will first be piloted with a small sample of study designs included in the review. The data will be extracted to prespecified and preapproved sheets using Microsoft Excel. These forms will be prespecified by the authors to include a recording of the study characteristics, the outcomes of interest and all relevant information. Furthermore, these forms will be shared with the author committee members for their feedback and for them to further pilot it put on a small number of studies. Based on this, the forms would either be revised or finalized. In this manner, these forms would be preapproved and finalized. The final version of the form will be used to extract and subsequently collect data from all included studies by the reviewers. Any disagreements will be resolved by the third reviewer.

If the data extracted are found to be limited, then we will contact the study authors for further information. Finally, we will also contact the authors of the included trials or studies to ascertain if they would like to answer any questions regarding the trials or studies. All the results will be presented in an appendix. Any missing or otherwise relevant information will be obtained from the primary authors of the original trials or studies (if needed).

Finally, in case of multiple publications, we will consider the final or the updated version of each study as the primary reference. This will ensure that the latest evidence is included in the review.

The following information will be extracted from the included studies:

- Training characteristics: we will extract characteristics such as the duration of training, frequency of training, the content of training, and location of training.
- The outcomes as reported by the study authors will include the following: (1) referral for diabetes, (2) screening for diabetes, (3) HbA_1c_ scores, and (4) fasting blood glucose values.

We will use the desktop version of software like Covidence (Veritas Health Innovation Ltd) or Sysrev (Insilica LLC) for the extraction process.

### Risk of Bias (Quality) Assessment

We will follow the revised risk of bias version 2 tool [37], which is a modified version of the Cochrane risk-of-bias tool to assess the internal validity of the included RCTs. The tool includes an assessment of sequence generation and allocation concealment; blinding of participants and outcome assessment; and incomplete outcome data and selective outcome reporting.

We will use ROBINS risk of bias tool in nonrandomized studies (if included) of intervention for assessing the risk of bias in nonrandomized studies included in the review. This will be done at the outcome level. All 3 reviewers (AGG, RD, and GRB) will be a part of the review. The first 2 reviewers will be conducting the review and in case of any disagreement which cannot be resolved between them, the third reviewer will resolve the disagreement and his decision will be final. If the information available from the sources is inadequate, then we will contact the primary authors of the studies to include details to request any missing data on items that could have contributed to the risk of bias. The individual bias items will be evaluated based on the items described in the *Cochrane Handbook for Systematic Review of Interventions* [38].

### Strategy for Data Synthesis

A preliminary search for studies showed a lot of heterogeneity, in terms of the training, like the study settings, participant education, professional experience, training frequency, training duration, and training content. Expecting this trend in the studies to be extracted and included, the authors will synthesize the training component narratively as per the suggestion in *Cochrane Handbook for Systematic Review of Interventions*.

The preliminary search also showed homogeneity in terms of the outcome results, like the mean change in fasting blood glucose values and the mean change in HbA_1c_ values. We will perform a meta-analysis and report the results in a PRISMA diagram [39].

The following are the data points which the reviewers would look for in synthesis. First, the effect of the intervention. Authors will determine if studies are estimating the same underlying treatment or intervention effect. As our review focuses on CHW training as the intervention, we will be looking if the studies are estimating the intervention effect in terms of knowledge, skills, and performance of the CHWs related to diabetes, that is, the interventions focus on screening, referral, HbA_1c_ levels, and fasting blood glucose levels. Additionally, the methodology and participants being studied will also be studied. Second, the factors affecting the implementation of the study interventions.

For the continuous data points, we would use the mean difference method for meta-analysis while we would use the odds ratio and the risk ratio methods for the discrete data points. In both cases, the confidence intervals would be 95%.

The narrative synthesis will be carried out by developing a general framework using a set of tools such as Microsoft Excel and Mendeley (Elsevier) or EndNote (Clarivate Analytics). This will be executed using the following steps:

- Develop a theory of change to understand how the intervention works, why it works, and for whom it best works.
- Development of preliminary synthesis of findings of all the studies to be included
- Exploration of relationships across the entire data from the included studies.
- Assessment of the robustness of the synthesis.

The main narrative findings will be reported in a table titled “Summary of Findings” by using the Grading of Recommendations, Assessment, Development and Evaluation approach as mentioned in the *Cochrane Handbook for Systematic Review of Interventions*. The within-study risk of bias to assess the methodological quality, the directness of the evidence, the heterogeneity, the precision effect estimates, and the risk of publication bias will also be considered.

Eventually, a rating system to rate the quality of the evidence for each of the outcomes in terms of high, moderate, low, and very low will be created. The same Grading of Recommendations, Assessment, Development and Evaluation method will also be used for the meta-analysis findings.

### Analysis of Subgroups or Subsets

We plan to perform subgroup analysis according to the following factors: (1) types of community health workers (nurses and lay health workers) and (2) demographic factors of health workers (education and work experience).

These factors will be analyzed for homogeneity or heterogeneity using the chosen software. All data would be entered in the software and tabulations would be carried out concerning each of the factors mentioned earlier to assess the heterogeneity or homogeneity. We would be using the Q statistic and I-square methods for assessing the heterogeneity. Further relationships will be explored within the data using the aforementioned factors and the predefined outcomes. A statistical test of significance will be carried out for each of the mentioned factors and an eventual summary will be presented to showcase statistical significance (if present) based on the *P* value of the association using the 1-tailed *t* test for each factor and its relationship with each outcome.

## Results

The review will start in May 2024 and will be completed in a time frame of 3 months. This will include the screening, abstract review, full-text review, data extraction, and data synthesis. The results are expected to be published before the end of 2024.

## Discussion

Diabetes is a pressing global health challenge, particularly affecting LMICs, where the prevalence is rapidly increasing among sections that are unable to afford or access quality health care. These patients are at high risk of developing complications which will affect the critical organs of the body. These combined with the delayed wound healing and their immunocompromised status, contributes to poorer outcomes for them. This underscores the importance of developing effective strategies for managing T2DM, particularly in LMICs where access to health care services are limited.

One such strategy is the use of CHWs for task shifting. CHWs, who are often members of the communities they serve, play a vital role in delivering health care services, including diabetes management in LMICs. However, CHWs lack the necessary knowledge and skills to manage diabetes and thus require training to improve their capacities. While there is some evidence to suggest that such training programs are successful in improving the CHWs knowledge, skills, and performance in diabetes management, such studies are few and their evaluations are rare. Therefore, there is a need for a systematic review to assess the impact of training programs for CHWs on diabetes outcomes in LMICs.

Possible findings from the review may include evidence supporting the effectiveness of training programs in improving CHWs’ ability to screen for diabetes, refer patients for further care, and manage diabetes-related outcomes such as HbA_1c_ levels and fasting blood glucose values. The review may also identify gaps in the existing literature, such as the lack of rigorous evaluations of training programs or the need for more studies in certain geographical regions or among specific populations.

The limitations of the study would be possible deviations from the protocols due to unexpected results in terms of number of studies, language restrictions, type and number of studies, and the quality of studies.

In conclusion, our protocol aims to establish a systematic review that will have the potential to influence future efforts of CHW training for effective diabetes management in LMICs.

## Appendix

Multimedia Appendix 1 List of Low-Middle-Income-Countries defined by World Bank 2024.

Multimedia Appendix 2 Provisional Search Strategy for PubMed.

Multimedia Appendix 3 PRISMA-P (Preferred Reporting Items for Systematic Reviews and Meta-Analyses–Protocol) checklist.

## Abbreviations

CHW: community health worker
HbA_1c_: hemoglobin A1c
LMIC: low- and middle-income country
PICOTS: Participants Intervention Comparator Outcome Time Study Design
PRISMA: Preferred Reporting Items for Systematic Reviews and Meta-Analyses
PRISMA-P: Preferred Reporting Items for Systematic Reviews and Meta-Analyses–Protocols
RCT: randomized controlled trial
T2DM: type 2 diabetes mellitus
WHO: World Health Organization

## Data Availability

Data sharing is not applicable to this article as no data sets were generated or analyzed during this study.

## References

[1] (2019). IDF Diabetes Atlas 9th Edition.

[2] Flood D, Seiglie JA, Dunn M, Tschida S, Theilmann M, Marcus ME, Brian G, Norov B, Mayige MT, Singh Gurung M, Aryal KK, Labadarios D, Dorobantu M, Silver BK, Bovet P, Adelin Jorgensen JM, Guwatudde D, Houehanou C, Andall-Brereton G, Quesnel-Crooks S, Sturua L, Farzadfar F, Saeedi Moghaddam S, Atun R, Vollmer S, Bärnighausen TW, Davies JI, Wexler DJ, Geldsetzer P, Rohloff P, Ramírez-Zea M, Heisler M, Manne-Goehler J (2021). The state of diabetes treatment coverage in 55 low-income and middle-income countries: a cross-sectional study of nationally representative, individual-level data in 680 102 adults. Lancet Healthy Longev.

[3] (2021). IDF Diabetes Atlas 10th Edition.

[4] Singh T, Nagesh S (2017). Magnitude and correlates of hypertension among geriatric women in a resettlement colony of Delhi. Int J Noncommun Dis.

[5] Vas P, Hopkins D, Feher M, Rubino F, B Whyte M (2020). Diabetes, obesity and COVID-19: a complex interplay. Diabetes Obes Metab.

[6] Mohan V, Anjana M, Pradeepa R, Unnikrishnan R, Kaur T, Das A, Muruganathan A, Bansode BR (2017). The ICMR INDIAB study—a compendium of type 2 diabetes in India: lessons learnt for the nation. Progress in Medicine 2017 (Medicine Update 2017).

[7] Rushforth B, McCrorie C, Glidewell L, Midgley E, Foy R (2016). Barriers to effective management of type 2 diabetes in primary care: qualitative systematic review. Br J Gen Pract.

[8] Jacobs B, Ir P, Bigdeli M, Annear PL, Van Damme W (2012). Addressing access barriers to health services: an analytical framework for selecting appropriate interventions in low-income Asian countries. Health Policy Plan.

[9] Long H, Huang W, Zheng P, Li J, Tao S, Tang S, Abdullah A (2018). Barriers and facilitators of engaging community health workers in non-communicable disease (NCD) prevention and control in China: a systematic review (2006⁻2016). Int J Environ Res Public Health.

[10] Lam AA, Lepe A, Wild SH, Jackson C (2021). Diabetes comorbidities in low- and middle-income countries: an umbrella review. J Glob Health.

[11] Liu J, Bai R, Chai Z, Cooper ME, Zimmet PZ, Zhang L (2022). Low- and middle-income countries demonstrate rapid growth of type 2 diabetes: an analysis based on Global Burden of Disease 1990–2019 data. Diabetologia.

[12] Mohan V, Anjana M, Pradeepa R, Unnikrishnan R, Kaur T, Das A (2017). Burden of Diabetes in INDIA the ICMR Indiab study-a compendium of type 2 diabetes in India: lessons learnt for the nation. Beyond Medicine.

[13] Thomas TA (2017). Diabetes is more prevalent among the urban poor: a summary of the findings of the ICMR-INDIAB study. Curr Med Issues.

[14] Mohan V, Sandeep S, Deepa R, Shah B, Varghese C (2007). Epidemiology of type 2 diabetes: Indian scenario. Indian J Med Res.

[15] Anjana RM, Deepa M, Pradeepa R, Mahanta J, Narain K, Das HK, Adhikari P, Rao PV, Saboo B, Kumar A, Bhansali A, John M, Luaia R, Reang T, Ningombam S, Jampa L, Budnah RO, Elangovan N, Subashini R, Venkatesan U, Unnikrishnan R, Das AK, Madhu SV, Ali MK, Pandey A, Dhaliwal RS, Kaur T, Swaminathan S, Mohan V (2017). Prevalence of diabetes and prediabetes in 15 states of India: results from the ICMR-INDIAB population-based cross-sectional study. Lancet Diabetes Endocrinol.

[16] Jeyashree K, Prinja S, Kumar MI, Thakur JS (2017). Inequity in access to inpatient healthcare services for non-communicable diseases in India and the role of out-of-pocket payments. Natl Med J India.

[17] Labhardt ND, Balo J, Ndam M, Grimm J, Manga E (2010). Task shifting to non-physician clinicians for integrated management of hypertension and diabetes in rural Cameroon: a programme assessment at two years. BMC Health Serv Res.

[18] Joshi R, Alim M, Kengne AP, Jan S, Maulik PK, Peiris D, Patel AA (2014). Task shifting for non-communicable disease management in low and middle income countries—a systematic review. PLoS One.

[19] Jeemon P, Narayanan G, Kondal D, Kahol K, Bharadwaj A, Purty A, Negi P, Ladhani S, Sanghvi J, Singh K, Kapoor D, Sobti N, Lall D, Manimunda S, Dwivedi S, Toteja G, Prabhakaran D (2016). Task shifting of frontline community health workers for cardiovascular risk reduction: design and rationale of a cluster randomised controlled trial (DISHA study) in India. BMC Public Health.

[20] Balasubramanya B, Isaac R, Philip S, Prashanth HR, Abraham P, Poobalan A, Thomas N, Jeyaseelan L, Mammen J, Devarasetty P, John O (2020). Task shifting to frontline community health workers for improved diabetes care in low-resource settings in India: a phase II non-randomized controlled clinical trial. J Glob Health Rep.

[21] Lekoubou A, Awah P, Fezeu L, Sobngwi E, Kengne AP (2010). Hypertension, diabetes mellitus and task shifting in their management in Sub-Saharan Africa. Int J Environ Res Public Health.

[22] Patel AR, Nowalk MP (2010). Expanding immunization coverage in rural India: a review of evidence for the role of community health workers. Vaccine.

[23] Khatri GR, Frieden TR (2002). Controlling tuberculosis in India. N Engl J Med.

[24] Warsi S, Elsey H, Boeckmann M, Noor M, Khan A, Barua D, Nasreen S, Huque S, Huque R, Khanal S, Shrestha P, Newell J, Dogar O, Siddiqi K (2019). Using behaviour change theory to train health workers on tobacco cessation support for tuberculosis patients: a mixed-methods study in Bangladesh, Nepal and Pakistan. BMC Health Serv Res.

[25] Lopes SC, Cabral AJ, de Sousa B (2014). Community health workers: to train or to restrain? A longitudinal survey to assess the impact of training community health workers in the Bolama region, Guinea-Bissau. Hum Resour Health.

[26] Abrahams-Gessel S, Denman CA, Montano CM, Gaziano TA, Levitt N, Rivera-Andrade A, Carrasco DM, Zulu J, Khanam MA, Puoane T (2015). The training and fieldwork experiences of community health workers conducting population-based, noninvasive screening for CVD in LMIC. Glob Heart.

[27] Bopape M, Mothiba T, Mutambudzi M, Wens J, Bastiaens H (2019). Baseline assessment of knowledge of home based carers for people with diabetes in a rural village in South Africa: a quantitative study. TOPHJ.

[28] Hughes GD, Puoane T, Bradley H (2014). Ability to manage diabetes—community health workers' knowledge, attitudes and beliefs. J Endocrinol Metab Diabetes S Afr.

[29] (2006). Definition and diagnosis of diabetes mellitus and intermediate hyperglycaemia: report of a WHO/IDF consultation. World Health Organization.

[30] WHO (2007). Community health workers: What do we know about them? the state of the evidence on programmes, activities, costs and impact on health outcomes of using community health workers. CHW Central.

[31] World Bank country and lending groups. The World Bank.

[32] Hongoro C, Normand C, Jamison DT, Breman JG, Measham AR, Alleyne G, Claeson M, Evans DB, Jha P, Mills A, Musgrove P (2006). Health workers: building and motivating the workforce. Disease Control Priorities in Developing Countries.

[33] Liang S, Deng H, Liu S, Wang G, Li L, Wang M, Pu J, Xing W, Luo X, Ehiri J, Xiang Y, Li Y (2019). Competency building for lay health workers is an intangible force driving basic public health services in Southwest China. BMC Health Serv Res.

[34] World Health Organization, PEPFAR, UNAIDS (2007). Task Shifting: Rational Redistribution of Tasks Among Health Workforce Teams: Global Recommendations and Guidelines.

[35] WMA resolution on task shifting from the medical profession. World Medical Association.

[36] PRISMA for systematic review protocols (PRISMA-P). PRISMA.

[37] Sterne JAC, Savović J, Page MJ, Elbers RG, Blencowe NS, Boutron I, Cates CJ, Cheng H, Corbett MS, Eldridge SM, Emberson JR, Hernán MA, Hopewell S, Hróbjartsson A, Junqueira DR, Jüni P, Kirkham JJ, Lasserson T, Li T, McAleenan A, Reeves BC, Shepperd S, Shrier I, Stewart LA, Tilling K, White IR, Whiting PF, Higgins JPT (2019). RoB 2: a revised tool for assessing risk of bias in randomised trials. BMJ.

[38] Higgins JPT, Thomas J, Chandler J, Cumpston M, Li T, Page MJ, Welch VA (2019). Cochrane Handbook for Systematic Reviews of Interventions.

[39] PRISMA flow diagram. PRISMA.

