# Supplementary material for: Training of Community Health Workers in Diabetes Lead to Improved Outcomes for Diabetes Screening and Management in Low- and Middle-Income Countries: Protocol for a Systematic Review
Source: JMIR Res Protoc. 2024 Aug 21;13:e57313. doi: 10.2196/57313 (PMC11375384; doi:10.2196/57313)
Supplement: Multimedia Appendix 1 [file resprot_v13i1e57313_app1.docx]

| Angola | Jordan | Philippines |
| --- | --- | --- |
| Algeria | India | Samoa |
| Bangladesh | Iran, Islamic Rep | São Tomé and Principe |
| Benin | Kenya | Senegal |
| Bhutan | Kiribati | Solomon Islands |
| Bolivia | Kyrgyz Republic | Sri Lanka |
| Cabo Verde | Lao PDR | Tanzania |
| Cambodia | Lebanon | Tajikistan |
| Cameroon | Lesotho | Timor-Leste |
| Comoros | Mauritania | Tunisia |
| Congo, Rep. | Micronesia, Fed. Sts. | Ukraine |
| Côte d'Ivoire | Mongolia | Uzbekistan |
| Djibouti | Morocco | Vanuatu |
| Egypt, Arab Rep. | Myanmar | Vietnam |
| Eswatini | Nepal | Zambia |
| Ghana | Nicaragua | Zimbabwe |
| Guinea | Nigeria |  |
| Haiti | Pakistan |  |
| Honduras | Papua New Guinea |  |
