# Supplementary material for: Training of Community Health Workers in Diabetes Lead to Improved Outcomes for Diabetes Screening and Management in Low- and Middle-Income Countries: Protocol for a Systematic Review
Source: JMIR Res Protoc. 2024 Aug 21;13:e57313. doi: 10.2196/57313 (PMC11375384; doi:10.2196/57313)
Supplement: Multimedia Appendix 2 [file resprot_v13i1e57313_app2.docx]

## **Appendix 2: Provisional Search Strategy for PubMed**

**((((((((((((((((((((((((((((((((((((((((((((((((((((((((((((((((((((((((((((((community health worker[MeSH Terms]) OR (community health workers[MeSH Terms])) OR (lay worker)) ) OR (CHW)) OR (health worker)) OR (health worker, community[MeSH Terms])) OR (health worker, village[MeSH Terms])) OR (health workers, community[MeSH Terms])) OR (ASHA)) OR (ANM)) OR (MPHW)) OR (JHA)) OR (MPW)) OR (MPHA)) AND (academic training[MeSH Terms])) OR (traini[MeSH Terms])) OR (activities, training[MeSH Terms])) OR (training)) OR (capacity building[MeSH Terms])) OR (activities, educational[MeSH Terms])) OR (activity, educational[MeSH Terms])) OR (education)) OR (hospital, teaching[MeSH Terms])) OR (hospitals, teaching[MeSH Terms])) OR (method, teaching[MeSH Terms])) OR (teaching)) OR (interactive tutorial[MeSH Terms])) OR (tutoring)) OR (coaching)) OR (supervision[MeSH Terms])) AND (effect, placebo[MeSH Terms])) OR (effects, placebo[MeSH Terms])) OR (placebo effect[MeSH Terms])) OR (placebos[MeSH Terms])) OR (placebo effects[MeSH Terms])) OR (care standard[MeSH Terms])) OR (care standards[MeSH Terms])) OR (standard training)) AND (type 2 diabetes mellitus[MeSH Terms])) OR (Typ[MeSH Terms])) OR (blood glucose[MeSH Terms])) OR (home blood glucose monitoring[MeSH Terms])) OR (monitoring, home blood glucose[MeSH Terms])) OR (Fasting Blood Glucose)) OR (FBS)) OR (Random Blood Glucose)) OR (Random Blood Sugar)) OR (Fasting Blood Sugar)) OR (RBS)) OR (consultation and referral[MeSH Terms])) OR (hospital referral[MeSH Terms])) OR (hospital referrals[MeSH Terms])) OR (referrals, hospital[MeSH Terms])) OR (referrals[MeSH Terms])) OR (physician self referral[MeSH Terms])) OR (physician self referrals[MeSH Terms])) OR (referrals, hospital[MeSH Terms])) OR (self referrals, physician[MeSH Terms])) OR (screening))) OR (a1b hemoglobin, glycosylated[MeSH Terms])) OR (glycosylated a1b hemoglobin[MeSH Terms])) OR (glycosylated hemoglobin[MeSH Terms])) OR (HbA1c)) OR (ogtt[MeSH Terms])) OR (glucose tolerance test[MeSH Terms])) OR (glucose tolerance tests[MeSH Terms])) OR (glucose tolerance, oral[MeSH Terms])) OR (microvascular complications)) OR (DR Screening)) OR (Diabetic Retinopathy Screening)) OR (Peripheral Neuropathy Screening) AND (clinicaltrial[Filter] OR meta-analysis[Filter] OR randomizedcontrolledtrial[Filter])) AND (countries, developing[MeSH Terms])) OR (countries, developing[MeSH Terms])) OR (countries, less developed[MeSH Terms])) OR (LMIC)) OR (Low and Middle Income Country)) OR (Low and Middle Income Countries)** Filters: **Clinical Trial, Clinical Trial, Phase III, Controlled Clinical Trial, Meta-Analysis, Pragmatic Clinical Trial, Randomized Controlled Trial, Systematic Review, Adult: 19+ years, from 2000/1/1 - 2023/4/1**
